# Acid-Induced Surface Degradation of Metallic Biomaterials: Alloy-Dependent Behavior and Implications for Surface Functionality

**DOI:** 10.3390/jfb17060268

**Published:** 2026-06-01

**Authors:** Réka Niklai, Péter Szabó, Judit Kopniczky, Tímea Dergez, Béla Kolarovszki, Orsolya Kada, Ákos Nagy, Kinga Turzó, Dorottya Frank

**Affiliations:** 1 Dental School, Faculty of Medicine, University of Pécs, Tüzér u. 1., H-7633 Pécs, Hungary; kolarovszki.bela@pte.hu (B.K.); orsi.kada@gmail.com (O.K.); nagy.akos@pte.hu (Á.N.); turzo.kinga@pte.hu (K.T.); frank.dorottya@pte.hu (D.F.); 2 Institute of Geography and Earth Sciences, University of Pécs, Ifjúság Útja 6., H-7624 Pécs, Hungary; sz.piiit01@gmail.com; 3 Department of Optics and Quantum Electronics, University of Szeged, Dóm Tér 9., H-6720 Szeged, Hungary; judit.kopniczky@gmail.com; 4 Institute of Bioanalysis, Medical School, University of Pécs, Honvéd u. 1., H-7624 Pécs, Hungary; timea.dergez@aok.pte.hu

**Keywords:** metallic biomaterials, corrosion, surface degradation, passive oxide layer, surface roughness, biocompatibility, surface functionality, ion release

## Abstract

Metallic biomaterials are frequently exposed to chemically aggressive environments that may compromise surface integrity and corrosion resistance. Acidic media containing organic acids represent a relevant challenge for metallic systems, as they can destabilize passive oxide layers and promote surface degradation processes. The present in vitro study investigated acid-induced surface alterations in four commercially relevant orthodontic alloys—nickel–titanium (NiTi), copper–nickel–titanium (CuNiTi), titanium–molybdenum alloy (TMA), and stainless steel—as representative metallic biomaterials. Specimens were exposed to two commercially available acidic beverages with distinct pH conditions, followed by analysis of surface morphology, roughness, and elemental composition using atomic force microscopy (AFM), scanning electron microscopy (SEM), and energy-dispersive X-ray spectroscopy (EDS). The results demonstrated pronounced alloy-dependent differences in degradation behavior. Stainless steel and TMAs exhibited significant increases in surface roughness and morphological alterations, whereas NiTi-based alloys showed comparatively stable surface characteristics. Elemental analysis revealed material-specific compositional variations, suggesting selective surface modification processes under acidic exposure. These differences can be attributed to variations in alloy composition, microstructure, and the stability of passive oxide layers, which collectively govern corrosion resistance in metallic systems. The findings provide insight into acid-induced degradation mechanisms in metallic biomaterials and highlight the importance of material-dependent corrosion behavior under chemically aggressive conditions. These observations may have implications for surface-mediated biological responses and long-term functional performance of metallic biomaterials.

## 1. Introduction

Energy drinks have gained substantial popularity since their introduction in the 1960s, particularly among adolescents and young adults [1]. These beverages are widely consumed to enhance alertness and physical performance and typically contain caffeine, taurine, B-group vitamins, and high sugar concentrations [2,3]. Epidemiological studies indicate that a considerable proportion of adolescents regularly consume energy drinks, making them a frequently consumed acidic beverage in this age group [4,5,6]. Reviews have also summarized their adverse systemic and metabolic effects [7,8].

Beyond their systemic effects, energy drinks create chemically aggressive acidic environments characterized by low pH and high titratable acidity [2,9]. Frequent exposure to such acidic media has been associated with dental erosion and material degradation processes [9,10,11,12,13]. In vitro studies have demonstrated that acidic beverages can significantly alter the surface properties of dental materials, increasing surface roughness and promoting degradation phenomena [10,11,12,13].

Acidic exposure is also relevant for metallic dental materials. Previous investigations have reported corrosion-related surface alterations and potential metal ion release from orthodontic components under acidic conditions [14,15,16]. These findings indicate that chemically aggressive environments may compromise surface integrity and stability of metallic systems. Recent studies have further demonstrated that acidic environments may significantly influence corrosion behavior, ion release dynamics, passive layer degradation, and oxide film stability of orthodontic biomaterials and titanium-based systems [17,18,19,20,21,22].

From a materials science perspective, the degradation behavior of metallic biomaterials is governed by alloy composition, microstructural characteristics, electrochemical environment, and the stability of passive oxide layers, particularly in titanium-containing systems [17,18,19,20,21,22,23,24]. Titanium-based biomaterials derive their corrosion resistance primarily from the formation of a stable TiO_2_ passive film, which may undergo localized degradation or destabilization under chemically aggressive or acidic conditions [17,18,19,20,21,22,23,24]. The protective oxide layer therefore plays a key role in surface stability and corrosion resistance; however, deterioration of this passive film may promote localized surface alterations, elemental redistribution, and corrosion-related degradation phenomena. Variations in alloy composition and microstructure may consequently result in material-specific degradation responses.

Orthodontic archwires represent metallic biomaterials that are continuously exposed to combined chemical and mechanical challenges in the oral environment. Surface integrity plays a crucial role in determining their mechanical performance, particularly with respect to frictional behavior and force transmission [25,26]. Even minor surface irregularities may influence sliding resistance and overall functional performance.

Therefore, the present in vitro study aimed to evaluate the influence of acidic beverage exposure on the surface morphology, roughness, and elemental composition of four commonly used orthodontic archwire alloys as representative metallic biomaterials. Surface alterations induced by acidic exposure may also influence biological interactions at the material interface, including protein adsorption, cellular responses, and ion release behavior, which are critical aspects of functional biomaterials.

## 2. Materials and Methods

### 2.1. Sample Preparation and Experimental Design

Four commercially available orthodontic rectangular archwire alloys (0.019″ × 0.025″) were investigated: copper–nickel–titanium (CuNiTi), nickel–titanium (NiTi), titanium–molybdenum alloy (TMA), and stainless steel (SS) (Rocky Mountain Orthodontics, Denver, CO, USA).

For each alloy, specimens were randomly allocated into three experimental groups (*n* = 7 per group per alloy; total *n* = 84 specimens across all alloys and groups): (I) Control (artificial saliva only), (II) Hell energy drink exposure, and (III) Burn energy drink exposure. Preformed archwires originating predominantly from the same manufacturing batch were sectioned into 10 mm segments. The sample size was selected based on comparable exploratory in vitro biomaterial surface characterization studies employing similar AFM, SEM, and EDS methodologies.

Each specimen was immersed individually in 100 mL of the respective energy drink under static conditions for 30 min at 37 °C. Following exposure, samples were rinsed with distilled water and subsequently immersed in artificial saliva for 30 min to simulate post-exposure neutralization. Control specimens were immersed in artificial saliva only under identical temperature conditions.

This experimental model was designed to simulate an acute acidic exposure event under standardized laboratory conditions and to evaluate early-stage surface degradation behavior of metallic biomaterials under controlled acidic exposure.

#### Rationale for the 30-Min Immersion Protocol

The 30 min immersion period was selected to approximate a typical energy drink consumption episode, during which orthodontic appliances may remain in direct contact with the acidic beverage for 15–30 min. Although clinical exposure is cyclic and repetitive, the present model was designed to simulate a single acute intraoral acidic challenge.

The subsequent 30 min immersion in artificial saliva was intended to approximate post-consumption salivary buffering and pH neutralization processes. This alternating exposure protocol (energy drink followed by artificial saliva) provides a standardized approach for assessing immediate surface alterations under controlled laboratory conditions while acknowledging that cumulative effects from repeated exposures may occur clinically.

### 2.2. Measurement of pH

The pH of each energy drink was measured using a Hanna HI 9321 microprocessor pH meter (Hanna Instruments Inc., Woonsocket, RI, USA). The electrode was calibrated with a pH 4.0 buffer solution prior to each measurement session.

Measurements were performed at 37 °C under static conditions without agitation. pH values were recorded at 5 min intervals over 30 min. Results are presented as mean ± standard error (SE).

### 2.3. Analysis of Surface Roughness and Morphology Using Atomic Force Microscopy (AFM)

Surface roughness and morphology were evaluated using a PSIA XE-100 atomic force microscope (PSIA Inc., Suwon, Republic of Korea) operating in tapping mode. Single-crystal silicon cantilevers (NSG30 series, NT-MDT, Moscow, Russia) were used.

For each specimen, six independent scan areas were recorded at two scan sizes (20 µm × 20 µm and 40 µm × 40 µm), generating technical replicates for each sample. Surface roughness (Ra) was calculated as the arithmetic mean deviation from the mean surface plane using the instrument AFM software version 1.5.

Identical scanning parameters were applied across all specimens to ensure consistency and comparability between groups.

### 2.4. Surface Morphology and Elemental Composition Analysis by SEM–EDS

Surface morphology and elemental composition were analyzed using a Jeol JSM-IT500HR scanning electron microscope (Jeol, Tokyo, Japan) equipped with an integrated energy-dispersive X-ray spectrometer (EDS).

Prior to SEM–EDS analysis, all specimens were sputter-coated with a thin gold layer (Jeol JFC-1300 Auto Fine Coater, Jeol, Tokyo, Japan) to improve electrical conductivity. SEM images were acquired in secondary electron imaging mode at accelerating voltages of 5 and 15 kV under high-vacuum conditions. Representative images presented in the manuscript were standardized at 1000× magnification for intergroup comparison.

For EDS analysis, three independent measurement points were obtained from each specimen and averaged for quantitative evaluation. Elemental compositions were expressed as atomic percentages (at%). During analysis, the contribution of the gold sputter coating was excluded to minimize analytical bias. Quantitative evaluation focused on relative changes in the principal alloy-forming elements. Identical coating parameters and acquisition settings were applied to all specimens to ensure valid comparison between control and treated groups.

### 2.5. Statistical Analysis

Normality of the datasets was assessed using the Shapiro–Wilk test. Since the surface roughness data showed a non-normal distribution, results are presented as the median and interquartile range (IQR). Group comparisons were performed using the Kruskal–Wallis test followed by post hoc pairwise comparisons where appropriate.

For pH measurements, data are presented as mean ± standard error (SE). A *p*-value < 0.05 was considered statistically significant. Statistical analyses were performed using SPSS software (version 21.0; IBM Corp., Chicago, IL, USA). An exploratory post hoc power analysis was performed for AFM-based surface roughness using an ANOVA-based approximation based on observed group means and standard deviations, acknowledging the exploratory nature of the present study.

## 3. Results

### 3.1. pH Characterization of Energy Drinks

Comparative pH analysis revealed distinct acidic profiles between the two commercially available energy drinks examined in this study. Both beverages demonstrated acidic pH values substantially below the critical threshold for enamel demineralization (pH 5.5). The Hell energy drink exhibited a mean pH of 3.59 ± 0.01, whereas Burn consistently showed lower pH values across all time points, with a pH of 2.81 ± 0.02.

Temporal pH stability was assessed through measurements conducted at 5 min intervals over 30 min, as presented in Table 1. The pH values remained relatively stable throughout the measurement period for both beverages, with Hell ranging from 3.53 to 3.62 and Burn ranging from 2.74 to 2.85. These findings confirm stable acidic conditions throughout the experimental exposure period.

### 3.2. Surface Roughness and Morphology by AFM

Atomic force microscopy was employed to quantitatively assess surface roughness (Ra) and qualitatively evaluate surface morphology of orthodontic archwires following exposure to energy drinks. Analysis was conducted at two distinct scan sizes (20 µm × 20 µm and 40 µm × 40 µm) to capture both fine-scale and larger-scale topographical features. In the AFM topographical images, surface elevation is represented through a color gradient, with lighter hues indicating elevated regions and darker brown tones representing depressed areas.

#### 3.2.1. CuNiTi Archwires

**Figure 1:**
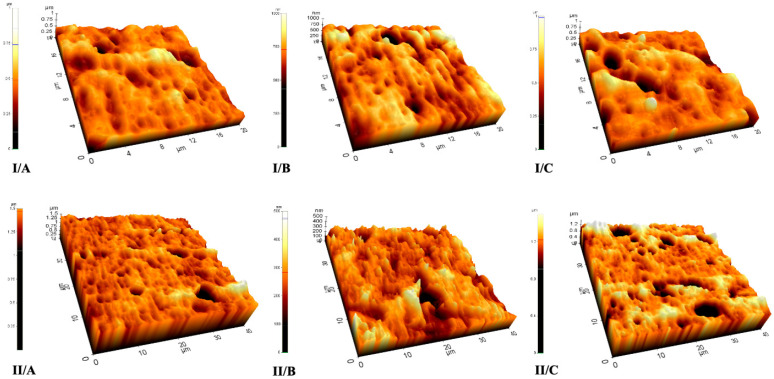
Atomic force microscopy topographical images of CuNiTi archwires. (**I/A**) Control specimen at 20 µm × 20 µm scan size; (**I/B**) Hell-treated specimen at 20 µm × 20 µm scan size; (**I/C**) Burn-treated specimen at 20 µm × 20 µm scan size; (**II/A**) Control specimen at 40 µm × 40 µm scan size; (**II/B**) Hell-treated specimen at 40 µm × 40 µm scan size; (**II/C**) Burn-treated specimen at 40 µm × 40 µm scan size.

**Figure 2:**
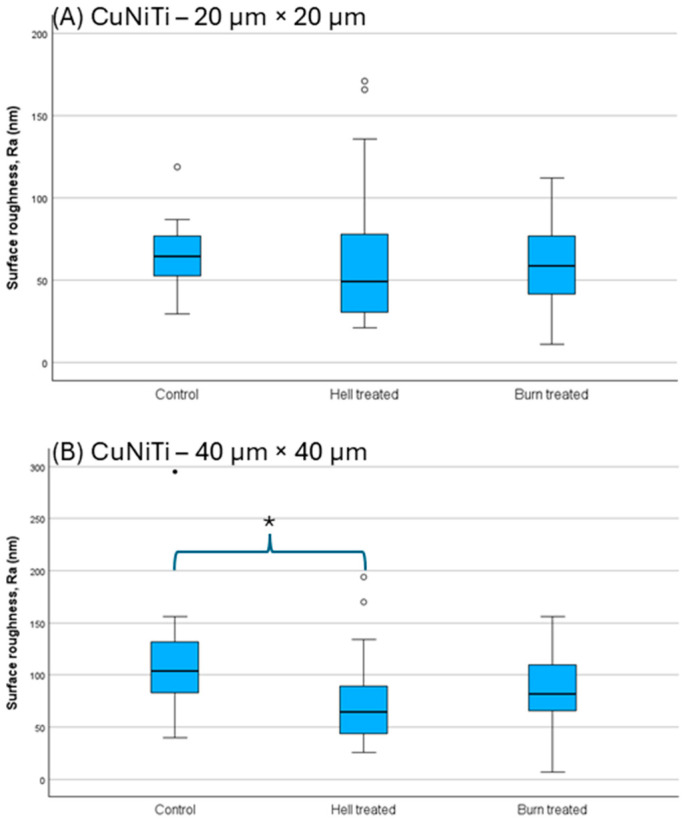
Atomic force microscopy (AFM)–based surface roughness (Ra, nm) of CuNiTi orthodontic archwires after exposure to energy drinks. Boxplots represent measurements obtained at two scan sizes: (**A**) 20 µm × 20 µm and (**B**) 40 µm × 40 µm. Boxes indicate the interquartile range (Q1–Q3), horizontal lines denote median values, and whiskers represent 1.5 × IQR. * indicates a statistically significant difference between Control and Hell-treated samples (*p* < 0.05, Kruskal–Wallis test with post hoc pairwise comparison).

Representative AFM topographical images of CuNiTi wire surfaces are presented in Figure 1. The surface morphology of untreated CuNiTi specimens characteristically displayed small surface voids interspersed with relatively symmetrical, rounded protrusions. Quantitative roughness data are illustrated in Figure 2.

At the 20 µm × 20 µm scan size, the untreated control CuNiTi samples exhibited a median surface roughness of 64.51 (52.00–78.50) nm (Figure 1I/A and Figure 2). Energy drink-treated specimens demonstrated modest reductions in Ra values compared to controls. Specifically, Hell-treated specimens yielded a roughness of 49.16 (30.70–78.06) nm (Figure 1I/B and Figure 2), while Burn-treated specimens measured 59.00 (41.83–80.00) nm (Figure 1I/C and Figure 2).

At higher spatial resolution, differences became more evident (40 µm × 40 µm). Control specimens demonstrated a surface roughness of 104.00 (82.00–132.25) nm (Figure 1 II/A and Figure 2). Both energy drink treatments produced statistically significant reductions in Ra values: Hell-treated specimens measured 64.53 (43.09–89.59) nm (Figure 1II/B and Figure 2) and Burn-treated specimens measured 81.50 (65.50–109.75) nm (Figure 1II/C and Figure 2). For CuNiTi wires at 40 µm × 40 µm scan size, pairwise comparison revealed that Hell-treated specimens exhibited significantly lower roughness compared to control specimens (*p* < 0.001).

#### 3.2.2. NiTi Archwires

**Figure 3:**
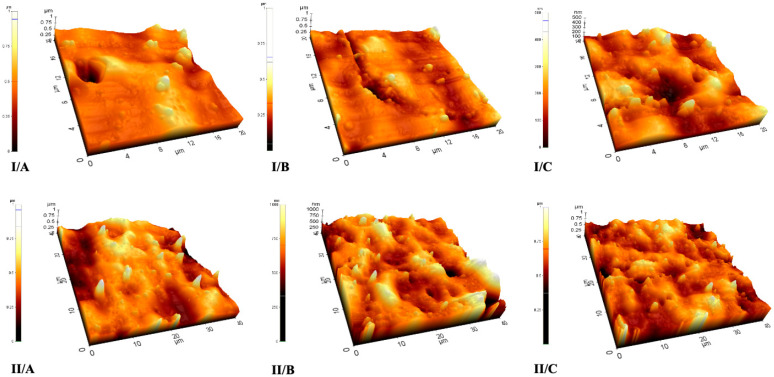
Atomic force microscopy topographical images of NiTi archwires. (**I/A**) Control specimen at 20 µm × 20 µm scan size; (**I/B**) Hell-treated specimen at 20 µm × 20 µm scan size; (**I/C**) Burn-treated specimen at 20 µm × 20 µm scan size; (**II/A**) Control specimen at 40 µm × 40 µm scan size; (**II/B**) Hell-treated specimen at 40 µm × 40 µm scan size; (**II/C**) Burn-treated specimen at 40 µm × 40 µm scan size.

**Figure 4:**
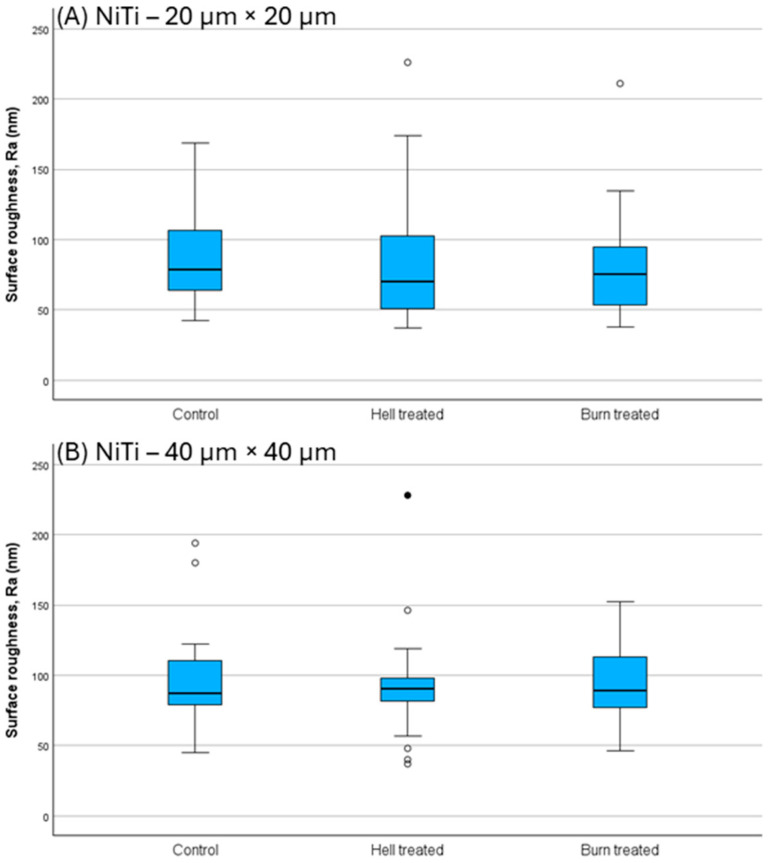
Atomic force microscopy (AFM)–based surface roughness (Ra, nm) of NiTi orthodontic archwires after exposure to energy drinks. Boxplots represent measurements obtained at two scan sizes: (**A**) 20 µm × 20 µm and (**B**) 40 µm × 40 µm. Boxes indicate the interquartile range (Q1–Q3), horizontal lines denote median values, and whiskers represent 1.5 × IQR. No statistically significant differences were observed between groups (Kruskal–Wallis test, *p* > 0.05).

Representative AFM images and quantitative roughness measurements for NiTi wire specimens are presented in Figure 3 and Figure 4, respectively.

In contrast to other alloys, NiTi wires exhibited remarkable stability. At a 20 µm × 20 µm scan size, control specimens demonstrated a median surface roughness of 78.50 (62.87–108.88) nm (Figure 3I/A and Figure 4). Energy drink-treated specimens exhibited comparable Ra values: Hell-treated specimens measured 70.50 (50.50–103.75) nm (Figure 3I/B and Figure 4), while Burn-treated specimens measured 75.37 (51.95–94.95) nm (Figure 3I/C and Figure 4).

Analysis at the 40 µm × 40 µm scan size yielded similar results. Control specimens exhibited a surface roughness of 87.00 (79.00–110.70) nm (Figure 3II/A and Figure 4). Hell-treated specimens demonstrated a roughness of 90.69 (81.21–98.62) nm (Figure 3II/B and Figure 4), and Burn-treated specimens measured 89.50 (77.00–116.50) nm (Figure 3II/ and Figure 4). Statistical analysis revealed no significant differences among the experimental groups at either scan size.

#### 3.2.3. TMA Archwires

**Figure 5:**
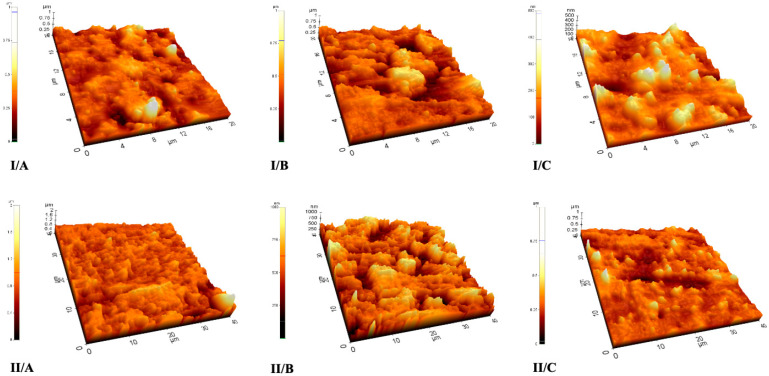
Atomic force microscopy topographical images of TMA archwires. (**I/A**) Control specimen at 20 µm × 20 µm scan size; (**I/B**) Hell-treated specimen at 20 µm × 20 µm scan size; (**I/C**) Burn-treated specimen at 20 µm × 20 µm scan size; (**II/A**) Control specimen at 40 µm × 40 µm scan size; (**II/B**) Hell-treated specimen at 40 µm × 40 µm scan size; (**II/C**) Burn-treated specimen at 40 µm × 40 µm scan size.

**Figure 6:**
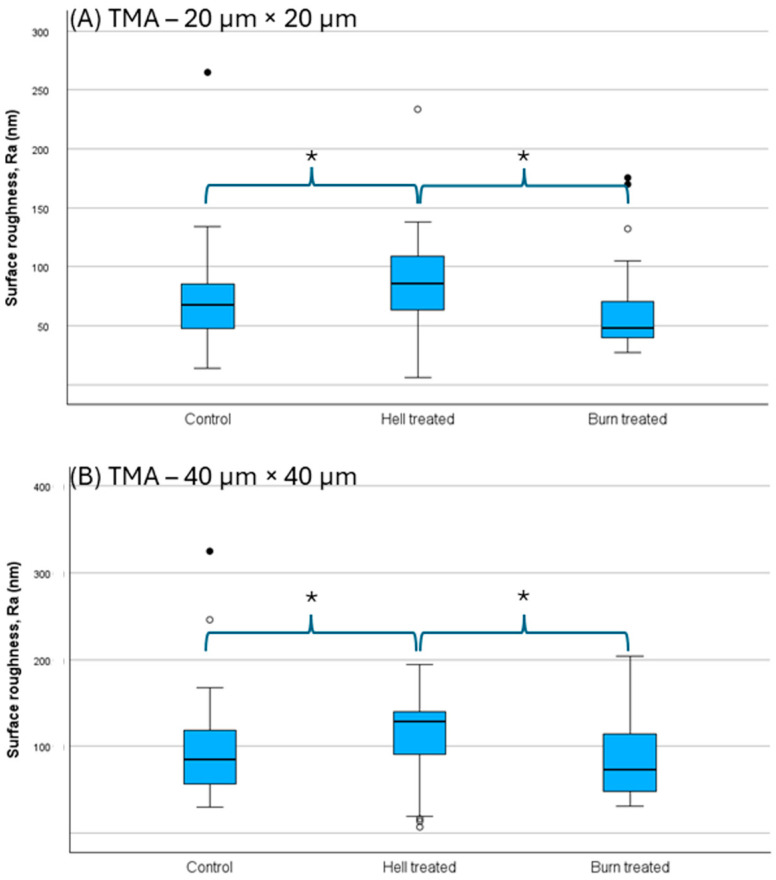
Atomic force microscopy (AFM)–based surface roughness (Ra, nm) of TMA orthodontic archwires after exposure to energy drinks. Boxplots represent measurements obtained at two scan sizes: (**A**) 20 µm × 20 µm and (**B**) 40 µm × 40 µm. Boxes indicate the interquartile range (Q1–Q3), horizontal lines denote median values, and whiskers represent 1.5 × IQR. * indicates statistically significant differences between groups (*p* < 0.05, Kruskal–Wallis test with post hoc pairwise comparison).

Atomic force microscopy analysis of TMA archwires revealed divergent surface responses to the two energy drink treatments, as illustrated in Figure 5 and Figure 6.

At the 20 µm × 20 µm scan size, untreated control specimens exhibited a median surface roughness of 68.05 (45.75–86.12) nm (Figure 5I/A and Figure 6). Hell energy drink treatment produced a statistically significant increase in surface roughness to 86.00 (61.74–109.11) nm (Figure 5I/B and Figure 6). Conversely, Burn energy drink treatment resulted in a modest reduction, yielding Ra values of 48.02 (39.62–71.53) nm (Figure 5I/C and Figure 6).

The opposing effects of the two energy drinks were similarly evident at the 40 µm × 40 µm scan size. Control specimens demonstrated Ra values of 85.02 (55.14–119.75) nm (Figure 5II/A and Figure 6). Hell treatment produced a significant elevation in surface roughness to 128.56 (89.50–141.75) nm (Figure 5II/B and Figure 6), while Burn treatment reduced roughness to 73.00 (47.71–123.25) nm (Figure 5II/C and Figure 6).

TMA wire analysis revealed significant differences at both scan sizes. Pairwise comparisons identified significant differences between control and Hell-treated specimens, as well as between Burn-treated and Hell-treated specimens. Hell treatment consistently increased surface roughness, while Burn treatment reduced roughness relative to the initial material state.

#### 3.2.4. SS Archwires

**Figure 7:**
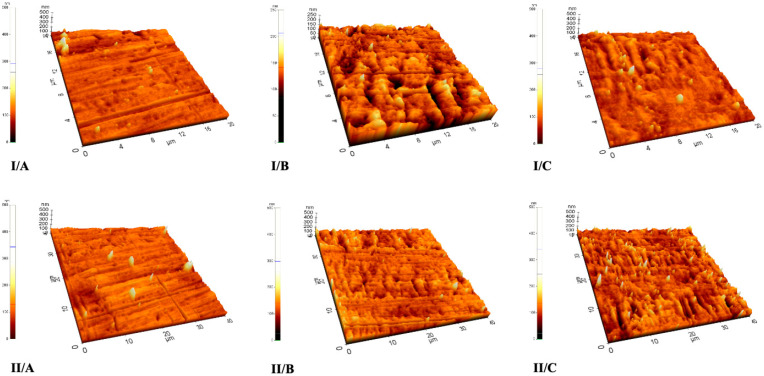
Atomic force microscopy topographical images of stainless steel archwires. (**I/A**) Control specimen at 20 µm × 20 µm scan size; (**I/B**) Hell-treated specimen at 20 µm × 20 µm scan size; (**I/C**) Burn-treated specimen at 20 µm × 20 µm scan size; (**II/A**) Control specimen at 40 µm × 40 µm scan size; (**II/B**) Hell-treated specimen at 40 µm × 40 µm scan size; (**II/C**) Burn-treated specimen at 40 µm × 40 µm scan size.

**Figure 8:**
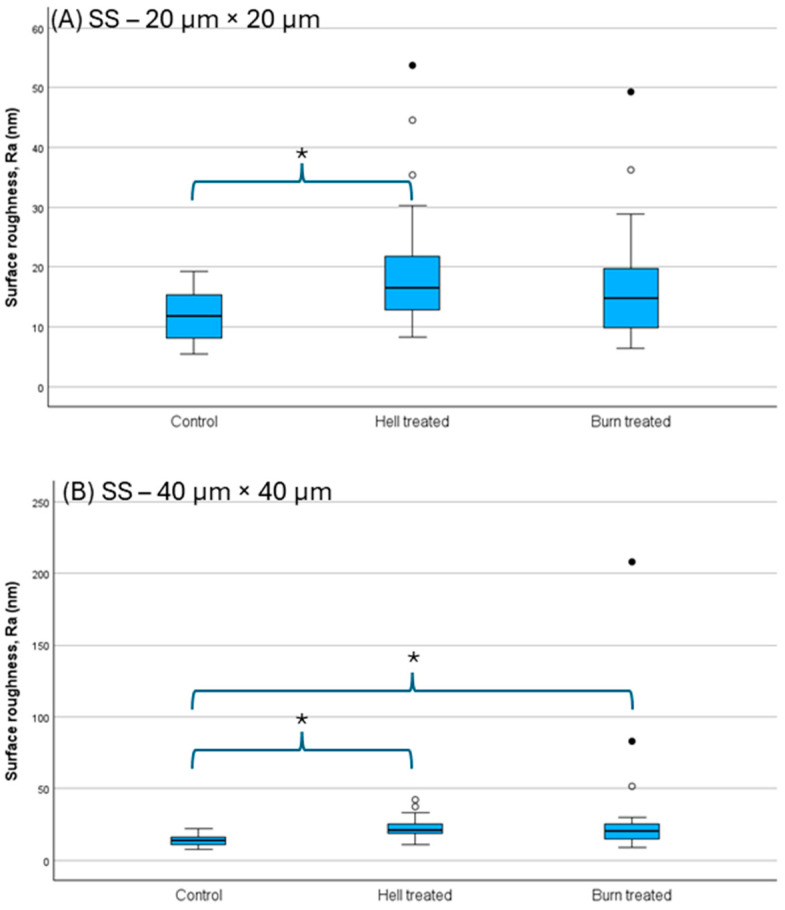
Atomic force microscopy (AFM)–based surface roughness (Ra, nm) of stainless steel (SS) orthodontic archwires after exposure to energy drinks. Boxplots represent measurements obtained at two scan sizes: (**A**) 20 µm × 20 µm and (**B**) 40 µm × 40 µm. Boxes indicate the interquartile range (Q1–Q3), horizontal lines denote median values, and whiskers represent 1.5 × IQR. * indicates statistically significant differences between groups (*p* < 0.05, Kruskal–Wallis test with post hoc pairwise comparison).

Stainless steel archwires demonstrated significant susceptibility to energy drink-induced surface alterations, as documented in Figure 7 and Figure 8.

At the 20 µm × 20 µm scan size, control specimens exhibited a baseline surface roughness of 11.83 (8.10–15.30) nm (Figure 7I/A and Figure 8). Hell treatment produced a statistically significant increase in Ra to 16.51 (12.57–22.30) nm (Figure 7I/B and Figure 8). Similarly, Burn treatment resulted in elevated roughness values of 14.86 (9.84–19.76) nm (Figure 7I/C and Figure 8), representing an approximate 50% increase relative to control specimens.

Analysis at the 40 µm × 40 µm scan size revealed even more pronounced effects. Control specimens demonstrated a roughness of 13.85 (10.97–16.24) nm (Figure 7II/A and Figure 8). Both energy drink treatments produced statistically significant increases: Hell-treated specimens measured 20.91 (18.88–26.48) nm (Figure 7II/B and Figure 8), while Burn-treated specimens exhibited the most substantial increase to 20.60 (14.99–25.69) nm (Figure 7II/C and Figure 8).

For stainless steel wires at the 20 µm × 20 µm scan size, Hell-treated specimens demonstrated significantly increased roughness compared to controls (*p* = 0.001). At the 40 µm × 40 µm scan size, both Hell-treated (*p* < 0.001) and Burn-treated (*p* < 0.001) specimens exhibited significantly elevated Ra values relative to controls. Overall, stainless steel demonstrated the greatest susceptibility to acidic surface alteration among the tested alloys.

### 3.3. Surface Morphology Analysis by SEM

Scanning electron microscopy provided high-resolution visualization of surface morphological changes following energy drink exposure, complementing the quantitative AFM data.

**Figure 9:**
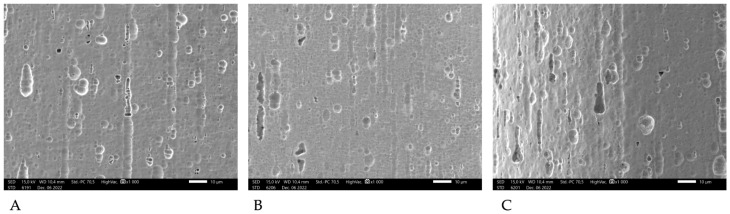
Scanning electron microscopy images of CuNiTi archwires at 1000× magnification. (**A**) Control specimen; (**B**) Hell-treated specimen; (**C**) Burn-treated specimen.

SEM examination of CuNiTi wires (Figure 9) corroborated the AFM findings, revealing the characteristic surface depressions observed in quantitative measurements. Following exposure to both Hell and Burn energy drinks, these surface voids appeared noticeably shallower compared to untreated controls, consistent with the observed reduction in surface roughness parameters.

**Figure 10:**
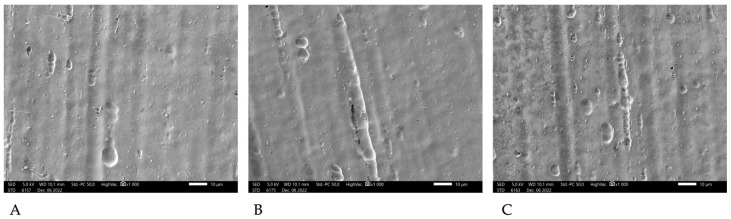
Scanning electron microscopy images of NiTi archwires at 1000× magnification. (**A**) Control specimen; (**B**) Hell-treated specimen; (**C**) Burn-treated specimen.

SEM analysis of NiTi specimens (Figure 10) revealed minimal morphological alterations following energy drink treatment. The surface topography maintained consistent characteristics across all groups, displaying smooth surfaces with shallow, rounded undulations. No discernible degradation or corrosion features were evident in treated specimens.

**Figure 11:**
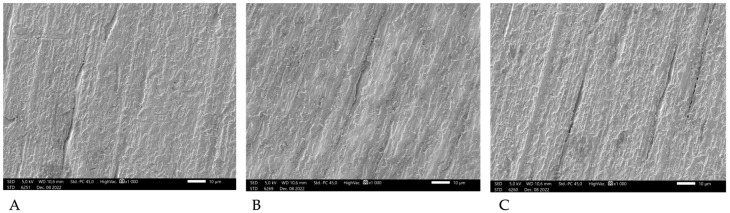
Scanning electron microscopy images of TMA archwires at 1000× magnification. (**A**) Control specimen; (**B**) Hell-treated specimen; (**C**) Burn-treated specimen.

High-magnification SEM imaging of TMA specimens (Figure 11) revealed the most pronounced morphological differences among the wire types examined. Hell-treated specimens displayed elevated and broadened surface protrusions, consistent with the increased roughness values measured by AFM. Conversely, Burn-treated specimens exhibited smoother surface characteristics with diminished topographical features, correlating with the decreased Ra values observed in quantitative analysis.

**Figure 12:**
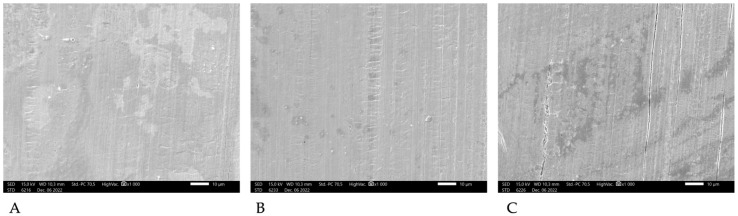
Scanning electron microscopy images of stainless steel archwires at 1000× magnification. (**A**) Control specimen; (**B**) Hell-treated specimen; (**C**) Burn-treated specimen.

SEM examination at 1000× magnification (Figure 12) revealed substantial surface degradation in energy drink-treated stainless steel specimens. Both Hell and Burn treatments produced deeper and more extensive surface cavities compared to untreated controls, with the morphological changes being clearly visible and consistent with the significant increases in surface roughness documented by AFM.

### 3.4. Surface Elemental Composition Analysis by EDS

Energy-dispersive X-ray spectroscopy was employed to assess alterations in surface elemental composition following energy drink exposure, providing insight into potential selective dissolution or surface enrichment phenomena.

#### 3.4.1. CuNiTi Archwires

**Figure 13:**
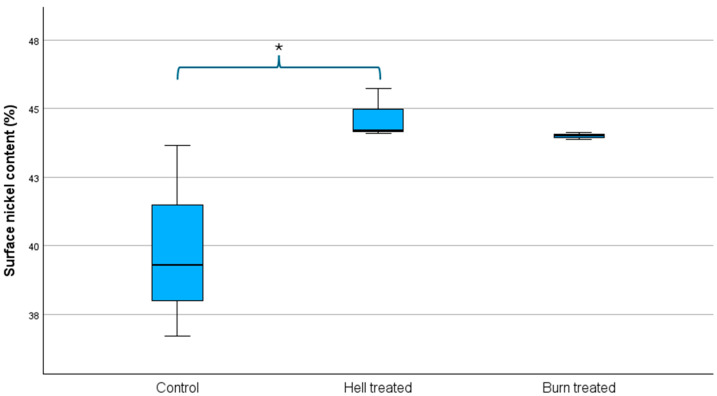
Boxplot representation of surface nickel content of CuNiTi orthodontic archwires determined by energy-dispersive X-ray spectroscopy (EDS). Boxes indicate the interquartile range (Q1–Q3), horizontal lines denote median values, and whiskers represent 1.5 × IQR. * indicates a statistically significant difference between Control and Hell-treated samples (*p* = 0.011, Kruskal–Wallis test with post hoc pairwise comparison).

EDS analysis of CuNiTi archwires revealed alterations in surface elemental composition following acidic exposure. A statistically significant increase in surface nickel proportion was observed in Hell-treated specimens compared to controls (*p* = 0.011), as illustrated in Figure 13. Quantitative elemental composition data presented in Table 2 demonstrated increased median nickel values in both acidic treatment groups relative to untreated controls. Minor variations in titanium and copper proportions were also observed following acidic exposure.

#### 3.4.2. NiTi Archwires

No statistically significant differences in surface elemental composition were detected between control, Hell-treated, and Burn-treated NiTi samples (*p* > 0.05). As summarized in Table 3, only minor variations in the relative proportions of titanium and nickel were observed following acidic exposure.

#### 3.4.3. TMA Archwires

**Figure 14:**
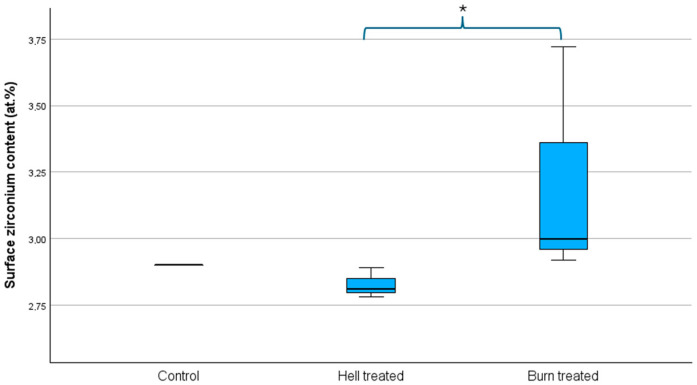
Boxplot representation of surface zirconium content of TMA orthodontic archwires determined by energy-dispersive X-ray spectroscopy (EDS). Boxes indicate the interquartile range (Q1–Q3), horizontal lines denote median values, and whiskers represent 1.5 × IQR. * indicates statistically significant differences between groups (*p* < 0.05, Kruskal–Wallis test with post hoc pairwise comparison).

Analysis of TMA archwires revealed statistically significant variations in surface zirconium content among experimental groups (*p* = 0.024). Pairwise comparison identified a significant difference between Burn-treated and Hell-treated specimens, with Burn treatment resulting in a higher surface zirconium proportion compared to Hell treatment (*p* = 0.006), as presented in Table 4 and Figure 14. In addition, quantitative EDS data demonstrated moderate variations in titanium, molybdenum, and tin proportions following acidic exposure.

#### 3.4.4. SS Archwires

EDS analysis of stainless steel archwires did not reveal statistically significant differences in surface elemental composition. Although Hell-treated specimens exhibited lower median nickel values compared to the other groups, this difference did not reach statistical significance (*p* = 0.051). As presented in Table 5, only minor variations in the relative proportions of the principal alloy-forming elements were observed following acidic exposure.

## 4. Discussion

The present study demonstrated that acidic exposure induces alloy-dependent degradation phenomena in metallic biomaterials, emphasizing the importance of environmental chemistry in corrosion-related processes. The observed alterations in roughness, morphology, and elemental composition indicate that even short-term acidic exposure may influence material stability in an alloy-specific manner.

Energy drinks represent chemically aggressive environments characterized not only by low pH but also by the presence of organic acids with potential chelating activity. These components may interact with metallic ions and contribute to destabilization of passive oxide layers, thereby promoting surface dissolution and electrochemically driven corrosion processes [2,9,17,18,19,20,21,22]. Such interactions may lead to topographical and compositional changes, particularly in multi-component alloys in which microstructural heterogeneity facilitates localized electrochemical activity [17,18,19,20,21,22,23,24].

The relative stability of nickel–titanium alloys may be attributed to the presence of a protective titanium oxide (TiO_2_) passive layer capable of providing effective corrosion resistance and rapid re-passivation following surface disruption [17,18,19,20,21,22,23,24]. The high chemical stability and self-repairing capacity of TiO_2_ likely contributed to the limited alterations observed in the NiTi groups.

In contrast, stainless steel specimens exhibited marked increases in roughness following acidic exposure. The corrosion resistance of stainless steel primarily depends on a chromium-rich passive layer, which may become less stable under strongly acidic conditions [15,16]. Disruption of this protective layer may result in localized heterogeneity and micro-pitting, thereby contributing to the roughness increases observed in the present study. The more pronounced alterations detected under lower pH conditions further support a pH-dependent degradation mechanism.

Titanium–molybdenum alloys demonstrated differential responses depending on the composition of the acidic medium, indicating that degradation behavior is influenced not only by pH but also by the specific chemical environment. The β-titanium microstructure of TMAs may contain compositional heterogeneities that promote micro-galvanic interactions under acidic conditions, resulting in localized variations in corrosion behavior and passive layer degradation [17,18,19,20,21,22,23,24]. In the present study, Burn exposure resulted in reduced roughness values relative to untreated controls. This phenomenon may reflect preferential dissolution of superficial asperities, resulting in partial nanoscale surface homogenization. Acidic exposure may additionally induce reorganization or partial reformation of the passive oxide layer, leading to smoother topographical characteristics despite ongoing corrosive interactions. Comparable smoothing effects following acidic exposure have previously been reported in investigations of metallic biomaterials [27].

SEM observations supported the AFM findings and confirmed the presence of alloy-dependent morphological alterations following acidic treatment. Surface irregularities, depressions, and localized defects were particularly evident in stainless steel and TMA specimens, whereas NiTi specimens maintained relatively stable characteristics. Since morphology is closely associated with frictional behavior, plaque accumulation, corrosion susceptibility, and long-term mechanical performance, these alterations may have clinical relevance during orthodontic treatment [25,26].

Elemental analysis revealed trends toward compositional redistribution, suggesting selective surface modification or enrichment phenomena. However, these findings should be interpreted cautiously. Although EDS analysis demonstrated alterations in elemental proportions following acidic exposure, this technique does not directly quantify ion release into the surrounding medium. Therefore, the observed compositional variations likely reflect localized dissolution and redistribution processes rather than direct evidence of metallic ion release. Previous investigations evaluating ion release from biomaterials have generally employed dedicated quantitative analytical techniques such as ICP-MS or atomic absorption spectroscopy [15,28,29,30].

The present investigation was designed as a standardized acute acidic exposure model to evaluate the initial alloy-dependent response under controlled experimental conditions. Although cyclic exposure protocols would more closely reproduce habitual energy drink consumption patterns, the use of a single continuous immersion protocol reduced the influence of confounding variables associated with cyclic models, including salivary buffering simulation, passive film regeneration, remineralization effects, and cumulative aging phenomena. This approach enabled direct comparison of the intrinsic susceptibility of the alloys investigated to acidic degradation under identical exposure conditions. Although the present model does not reproduce chronic or repeated exposure, it was designed to simulate a standardized acute acidic challenge corresponding to a typical energy drink consumption episode. The observation that measurable alterations occurred after a single exposure suggests that repeated consumption may result in cumulative changes, particularly in more susceptible alloys. Therefore, the clinical relevance of the present study lies in identifying alloy-dependent early vulnerability under acidic conditions rather than directly modeling long-term intraoral exposure. Furthermore, prolonged immersion protocols are commonly used in in vitro biomaterial corrosion studies as accelerated screening approaches for evaluating early degradation phenomena and material-dependent behavior [10,15,27,28].

From a functional biomaterials perspective, corrosive changes and compositional alterations may influence protein adsorption, cellular interactions, and potential ion release behavior. Surface topography and chemistry are known to play important roles in mediating biological responses, including cell adhesion and biofilm formation [31]. Although these aspects were not directly investigated in the present study, the observed modifications may influence biological interactions at the biomaterial interface and potentially affect long-term functional performance.

More broadly, the present findings emphasize that corrosion behavior in metallic biomaterials is governed by the interplay between environmental conditions and intrinsic material characteristics, including alloy composition and passive layer stability [32]. Although the present study employed an acute exposure model, repeated acidic challenges may result in cumulative material alterations over time.

Several limitations should be acknowledged. The in vitro design does not account for repeated cyclic exposure, dynamic electrochemical conditions, tribocorrosion effects, salivary fluctuations, or long-term degradation kinetics. Furthermore, electrochemical measurements and direct ion release analyses were not performed, limiting detailed mechanistic interpretation of the corrosion behavior. Electrochemical techniques such as potentiodynamic polarization testing and electrochemical impedance spectroscopy (EIS) could provide additional quantitative insight regarding corrosion kinetics, pitting susceptibility, and repassivation behavior. Future studies integrating electrochemical analyses, cyclic acidic exposure protocols, artificial aging models, tribological approaches, and biological assays may provide deeper insight into degradation mechanisms and functional performance under clinically relevant conditions.

Overall, the present findings demonstrate that alloy composition and passive layer characteristics play a critical role in determining susceptibility to acid-induced degradation in metallic biomaterials.

## 5. Conclusions

Within the limitations of this in vitro study, acidic exposure induced material-dependent surface degradation in metallic orthodontic alloys, demonstrating that corrosion behavior is strongly influenced by alloy composition and passive oxide layer stability.

Nickel–titanium alloys exhibited superior resistance to surface alteration, whereas stainless steel and titanium–molybdenum alloys showed increased susceptibility to roughness changes under acidic conditions, reflecting differences in passive layer stability and microstructural characteristics.

The present results provide insight into corrosion-related surface modification processes in metallic biomaterials exposed to acidic environments and highlight the importance of material-dependent degradation behavior. These findings may also be relevant for understanding surface-mediated biological interactions and the functional performance of metallic biomaterials in chemically aggressive conditions.

## Figures and Tables

**Table 1:** Temporal pH measurements of Hell and Burn energy drinks recorded at 5 min intervals over a 30 min period. Data presented as mean pH ± standard error (SE).

Time (Minutes)	0	5	10	15	20	25	30	Mean pH ± SE
Hell	3.61	3.62	3.62	3.53	3.59	3.57	3.59	3.59 ± 0.01
Burn	2.85	2.85	2.84	2.76	2.83	2.82	2.74	2.81 ± 0.02

**Table 2:** Surface elemental composition of CuNiTi orthodontic archwires determined by energy-dispersive X-ray spectroscopy (EDS). Data are presented as median (interquartile range). n.a. = not applicable.

Element	Control Median (IQR)	Hell Treated Median (IQR)	Burn Treated Median (IQR)	*p*-Value
Ni	39.31 (38.02–41.48)	44.20 (44.14–44.97)	44.01 (43.95–44.08)	0.039
Ti	45.79 (44.51–48.79)	51.35 (51.26–52.81)	51.29 (51.26–51.32)	0.491
Cu	4.15 (3.99–13.33)	4.60 (4.56–4.64)	4.76 (4.64–4.79)	0.236
O	10.74 (5.37–13.33)	n.a.	n.a.	n.a.
Al	0.00 (0.00–0.17)	n.a.	n.a.	n.a.

**Table 3:** Surface elemental composition of NiTi orthodontic archwires determined by energy-dispersive X-ray spectroscopy (EDS). Data are presented as median (interquartile range). n.a. = not applicable.

Element	Control Median (IQR)	Hell Treated Median (IQR)	Burn Treated Median (IQR)	*p*-Value
O	16.04 (8.02–17.08)	0.00 (0.00–9.53)	n.a.	0.817
Al	0.51 (0.44–0.57)	0.00 (0.00–0.20)	0.00 (0.00–0.18)	0.141
Ti	43.05 (42.33–46.99)	50.81 (45.99–51.05)	50.56 (50.52–50.73)	0.875
Ni	40.54 (40.22–44.49)	48.72 (44.06–48.96)	48.90 (48.85–49.17)	0.148

**Table 4:** Surface elemental composition of TMA orthodontic archwires determined by energy-dispersive X-ray spectroscopy (EDS). Data are presented as median (interquartile range).

Element	Control Median (IQR)	Hell Treated Median (IQR)	Burn Treated Median (IQR)	*p*-Value
O	26.06 (25.64–26.12)	26.49 (25.25–28.58)	22.40 (21.37–23.43)	0.061
Ti	64.26 (64.22–64.58)	64.07 (62.18–65.13)	68.26 (67.41–77.67)	0.061
Zr	2.90 (2.90–2.90)	2.81 (2.79–2.85)	3.00 (2.96–3.36)	0.024
Mo	5.15 (5.13–5.17)	4.94 (4.79–5.16)	5.36 (5.12–6.14)	0.670
Sn	1.65 (1.61–1.74)	1.62 (1.61–1.65)	2.29 (2.02–3.32)	0.113

**Table 5:** Surface elemental composition of SS orthodontic archwires determined by energy-dispersive X-ray spectroscopy (EDS). Data are presented as median (interquartile range).

Element	Control Median (IQR)	Hell Treated Median (IQR)	Burn Treated Median (IQR)	*p*-Value
Si	1.34 (1.32–1.38)	1.33 (1.32–1.40)	1.44 (1.39–1.46)	0.494
Cr	20.38 (20.34–20.60)	20.41 (20.25–20.42)	20.37 (20.32–20.49)	0.875
Mn	1.12 (1.09–1.20)	1.25 (1.23–1.32)	1.23 (1.22–1.36)	0.432
Fe	68.66 (68.62–68.74)	69.03 (68.88–69.04)	68.74 (68.57–68.83)	0.288
Ni	8.07 (8.06–8.09)	7.98 (7.91–8.00)	8.05 (8.04–8.08)	0.051
Al	0.30 (0.15–0.35)	0.00 (0.00–0.25)	0.00 (0.00–0.19)	0.808

## Data Availability

The raw data supporting the conclusions of this article will be made available by the authors on request.
